# Influence of Feeding Compound Feed Rich in Fibre during Parturition and Lactation on Health and Performance of Sows

**DOI:** 10.3390/ani12040497

**Published:** 2022-02-17

**Authors:** Cornelia Schwennen, Bernd Reckels, Maria Klingenberg, Amr Abd El-Wahab, Birgit Keller, Christian Visscher

**Affiliations:** 1 Institute for Animal Nutrition, University of Veterinary Medicine Hanover, Foundation, 30173 Hanover, Germany; sekretariat-tierernaehrung@tiho-hannover.de (M.K.); amrwahab5@mans.edu.eg (A.A.E.-W.); service-tierernaehrung@tiho-hannover.de (B.K.); christian.visscher@tiho-hannover.de (C.V.); 2 Department of Nutrition and Nutritional Deficiency Diseases, Faculty of Veterinary Medicine, Mansoura University, Mansoura 35516, Egypt

**Keywords:** *C. perfringens*, pigs, dietary fibre, feed intake, lactation, fecal quality, transition period

## Abstract

**Simple Summary:**

Although it is known that restriction of feed negatively influences the behaviour and welfare of sows, it is widely adopted during the gestation period to counteract nutrient oversupply. Nonetheless, a healthy gut needs its fuel and is a prerequisite for preventing birth-associated health disorders. Including high amounts of fibre in diets around parturition could be the solution for behavioural disorders and health problems in sows and therefore in piglets as well. The purpose of this study was to investigate the influence of ad libitum access to compound feeds rich in fibre (ante partum and peri partum) and a lactation diet (post partum) on the performance and health of sows. This study indicated that a higher feed intake of a fibre-rich diet ante partum did not interfere with any birth-associated disorders. In addition, ad libitum-fed sows showed higher dry matter (DM) intake throughout lactation, which led to beneficial effects on the body condition scores of sows. Due to the high fibre intake, the excretion of *C. perfringens* via sows’ faeces could be significantly reduced, which could be a helpful tool in reducing the risk of neonatal diarrhoea caused by *C. perfringens*.

**Abstract:**

The aim of this study was to investigate the influence of ad libitum access to compound feeds rich in fibre (ante partum (a.p.) and peri partum) on the DM intake, body mass development and performance of sows as well as excretion of *Clostridium* (*C.*) *perfringens* via sows’ faeces. From day 109 (d-7) of gestation, 25 of 48 sows (23 considered as control) received access to one of two different high-fibre pellets from d-7 until the second day post partum (p.p.) (d2) (fibre groups (FG) 1 and 2) in additional to a lactation diet. The additional DM intake of the high-fibre pellets a.p. was 2.13 ± 1.15 kg in FG 1 and 3.14 ± 0.68 kg in FG 2. This led to higher DM intake in the first lactation week and significantly lower losses of weight and back fat thickness during lactation. The bacterial counts of *C. perfringens* in sows’ faeces directly p.p. were 10 times lower in FG 1 and 100 times lower in FG 2 compared to the controls. High amounts of fibre led to higher DM intake throughout lactation, which had beneficial effects on sows’ body conditions. It seems that high fibre intake influenced the excretion of *C. perfringens* at parturition, which could improve the health of newborns.

## 1. Introduction

An adequate supply of modern high-performance sows with energy and nutrients, especially during the lactation period, is still a challenge in animal nutrition [1]. Once sows have entered the farrowing pen, they are usually fed a restrictive low-fibre, concentrated lactation diet until giving birth, due to old fears that high feed intake ante partum is associated with birth disorders [2,3]. Although the allocated feed quantity is gradually increased after birth and the lactation diet is offered nearly ad libitum from the second week of lactation onwards, a low feed intake in the first week of lactation cannot be compensated by a higher feed intake in the subsequent lactation [4]. This leads to a reduced overall feed intake during the lactation period accompanied by weight loss and may result in several common reproductive problems, such as extended weaning-to-oestrus intervals [5]. The transition period is defined as the last 10 d of gestation to the first 10 d of lactation [6] and is a crucial phase for the sow and offspring as well [7]. Especially in the last weeks of gestation, not only do the strongest growth of foetuses and massive mammary growth take place, but also the production of colostrum [8,9,10]. Therefore, supplementation of extra nutrients and energy during this period is important to improve the performance of sows and piglets as well during farrowing and lactation [8,11,12,13]. Apart from that, the role of fibre in sow nutrition during gestation and early lactation has received more and more interest. Several studies have shown that high-fibre diets can beneficially influence the behaviour and welfare of sows [14,15,16,17] as well as their reproductive performance [18,19,20]. Including high amounts of fibre in gestation diets is not only a method for promoting satiety [21] and reducing stereotypical behaviour [14] but also for increasing voluntary feed intake especially during early lactation [19,20].

The prenatal and postnatal phases are crucial periods for the development of the intestinal microbiota and immune systems of offspring. It is believed that the gastrointestinal tract of newborn piglets in the uterus is sterile and that colonization with microorganisms starts after birth [22]. Maternal microbiota influence offspring gut colonization in the first hours of life through direct contact with maternal faeces during birth and through colostrum intake [23]. For example, neonatal suckling pigs are primarily infected by *Clostridia* ssp. through sow faeces. Clostridial enteric infections are common in pig husbandry and may lead to high losses, especially in suckling piglets [24]. In this context, *C. perfringens* type C and *C. difficile* are the main pathogens found in enteric disease outbreaks [25]. However, diagnosis of neonatal piglet diarrhoea due to *C. perfringens* type A has increased in clinical cases where no other enteric pathogen could be found [24,26]. If maternal colostrum, milk quality and microbiota composition are influenced by diets, maternal dietary manipulation could be an effective way to improve offspring’s health. Thus, previous studies have shown that probiotic and prebiotic supplementation during gestation and lactation could improve the quality of colostrum and milk, modulate gut microbiota and stimulate the development of intestinal immunity in newborns [27,28]. As previously mentioned, results of research demonstrated the beneficial impact of fibre-rich diets on the behaviour, welfare and performance of sows [14,15,16,17,18,19,20]. However, only a few studies have investigated the effect of maternal fibre nutrition in gestation and early lactation on the (gut) health and behaviour of newborn piglets [29,30].

The aim of this project was to investigate the influence of ad libitum compound feeds rich in fibre (ante partum and peri partum) and a lactation diet (post partum) on the DM intake, body mass development and performance of the sows during lactation, and the excretion of *C. perfringens* via the sow faeces. Therefore, the hypothesis of the study was that feed intake post partum and the excretion of *C. perfringens* can be positively influenced by a compound feed rich in fibre offered ad libitum during the transition period. For this purpose, two differently composed compound feeds rich in fibre (fibre pellet 1 and fibre pellet 2) were used alternately.

## 2. Materials and Methods

### 2.1. Sows and Management

The study took place in the farrowing stable of the farm for education and research at the University of Veterinary Medicine Hannover, Foundation, in Hanover, Germany. This conventionally managed farm housed 80 BHZP sows and worked in a two-week rhythm with a 35-day suckling period. For study purposes, the sows were moved to two identical farrowing units (with a base area of 2.28 m × 1.98 m) one week prior to the calculated date of birth. The sows were routinely vaccinated with the combination vaccine against *Erysipelothrix rhusiopathiae* and Porcine Parvovirus (PPV) (Parvoruvac^®^; Ceva Tiergesundheit GmbH; Düsseldorf, Germany), also receiving an active immunisation for the passive immunisation of piglets against neonatal enterotoxicosis due to *E. coli* (Porcilis^®^ Porcoli Diluvac Forte; Intervet Deutschland GmbH; Unterschleißheim, Germany). Each farrowing unit consisted of eight farrowing pens divided through an inspection walkway. The farrowing pen was equipped with a farrowing crate placed at an angle to the inspection walkway, a heated piglet nest closed at the top and a nipple drinker for the piglets. During the entire test phase, a temperature/humidity logger (EBI 20 series; WTW GmbH; Ebro Electronic Business Unit; Ingolstadt, Germany) was installed in the animal area in order to measure the temperature and the humidity.

### 2.2. Study Design and Feeding of the Sows

A total of seven rounds with a maximum of eight animals each were examined, each round consisting of a control group and a fibre group. The animals in the control group were exclusively fed the lactation diet in accordance with the feed curve (control group (CG) 1 (n = 10) or 2 (n = 13)), while the fibre groups were given the corresponding fibre pellet feed (pellet 1 or 2) and then the lactation diet ad libitum (fibre group (FG) 1 (n = 12) or 2 (n = 13)). When the sows were placed in the farrowing pen, the feed was changed from a complete pelleted feed for pregnant sows (FESONI-FEG NT 11.8 K Ruthe; Bruno Fehse und Sohn GmbH & Co. KG; Estorf-Leeseringen, Germany) to a complete pelleted feed for lactating sows (FESONI-FEG ZS Lac 13.0 K Ruthe; Bruno Fehse und Sohn GmbH & Co. KG) (Table 1).

The restrictive lactation diet was fed twice a day via a volume feeder. From the time of entering the farrowing unit (d-7) up until day one after giving birth, the sows received 1.4 kg DM per meal of the lactation diet. From the second day post partum (p.p.), the amount of feed was increased stepwise, depending on the number of piglets, by about 0.5 kg per day so that the sows were fed, according to their individual maximum feed intake capacities, almost ad libitum after the first week of lactation. The animals in the trial groups (fibre groups 1 and 2) were given access to an extra feeding dispenser above their troughs, filled from d-7 until the second day after giving birth (d2 p.p.) with one of the fibre pellets (Table 2) in addition to the restrictively fed lactation diet.

The chemical composition of the lactation diet and the fibre pellets 1 and 2 were analysed at the Institute of Animal Nutrition (University of Veterinary Medicine Hannover, Foundation). The investigations were carried out in a double approach in accordance with the official methods of the VDLUFA (Association of German Agricultural Analytic and Research Institutes) [31] with modifications made by the Institute itself [32] (Table 3). The dry matter content was determined by drying to the weight constancy at 103 °C.

**Figure 1:**
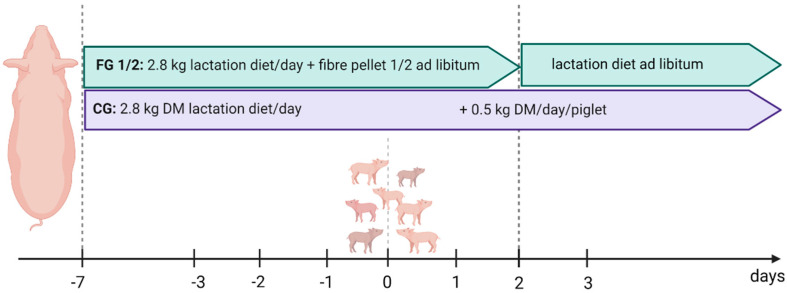
Feeding scheme of the trial and control groups during the experimental phase (figure was created with BioRender.com, accessed on 23 December 2021).

To improve the acceptance of fibre pellet 1 and 2, both were mixed with 20% of the commercial lactation diet. From d3 p.p. onwards, only the lactation diet was filled into the extra feeding dispensers (Figure 1) in the experimental groups. The sows had unlimited access to water via cone drinkers (flow rate ≥4 L/min) mounted in the trough.

### 2.3. Parameters

#### 2.3.1. Feed Intake Capacity

To record individual daily feed intake, the content of each volume feeder was reweighed manually after every new adjustment. In the trial groups, the amount of fibre pellets filled into the feed dispensers was weighed every morning. In the case of decreased appetite or spoiled feed in the trough, refusals were removed from the trough and weighed back. The DM was determined, and the loss was included in the calculation of the daily amount of feed intake (in kg DM). However, not all sows farrowed on exactly the same day, so each sow had already been in the farrowing pen for different lengths of time at the time of birth. As the day of birth was set as day 0, a different number of sows were included in the statistical evaluation for days −6 to −3-depending on the farrowing day of each individual sow.

#### 2.3.2. Parameters of Birth and Performance of the Sows

Birth monitoring was carried out between 06:00 and 00:00. Sows that farrowed during the unobserved period were not included in the evaluation of birth parameters. Observations included duration of farrowing (i.e., the time from first- to last-born piglet) and the average time between the farrowing of two piglets, as well as the number of piglets born alive, dead and mummified. Interventions (obstetrical or medicinal) were also measured. In addition, piglet losses and the causes of these losses—as far as known—were recorded for each sow on d1, d2 and d3 and after d4. Furthermore, the number of weaned piglets per sow was noticed.

#### 2.3.3. Cross-Fostering

Due to the high number of piglets born alive, cross-fostering was necessary for enhancing the welfare of the piglets. If the building of foster mothers was necessary from sows participating in the trials, the data from these sows were excluded from the data set.

#### 2.3.4. Body Mass and Backfat Thickness of the Sows

The sows were weighed at four different times during the trial, always in the morning two hours after feeding. The first time was one week prior to the calculated date of birth (d-7), then 24 h p.p.—after the first feed intake—, at the 14th day of lactation (d14) and, for the last time, at the day of leaving the farrowing unit (d35 p.p.). The ground-level scales (Soehnle Industrial Solutions GmbH; Backnang, Germany) were located on the central supply corridor in front of the farrowing units, so that the sows had to leave the farrowing pen for a short time for weighing.

Parallel to the weighing, the backfat thickness of the sows was measured by ultrasound examination (Lean-Meater^®^; Renco; Golden Valley, MT, USA). The backfat above *M. longissimus* is composed of three layers of tissue: skin (cutis) and first (subcutis) and second layers of fat (interfascial fat layer), as well as the connective tissue layer (fascia lumbodorsalis) between the interfascial fat layer of the back fat and the back muscle. In accordance with Müller and Polten [35], the thickness of backfat was measured at three points along the back, each 6 cm lateral (paramedian) to the spine. A mean value was then formed from the three measured values.

#### 2.3.5. Faecal Analysis

The consistency of the sows’ faeces was assessed by a scoring system [17] at least twice a week during the trial period and daily from d-2 to one week after birth (d7). The score value ranged from 0 to 5, 0 indicating the absence of faeces and 5 diarrhoea (Table 4).

During the trial period, the pH value was determined in a total of 10 faecal samples per sow. For this purpose, rectal faecal samples were taken from the sows at entering the farrowing pen, every two days during the farrowing week and weekly from the second week of lactation onwards. To determine the pH value, approx. 1 g faeces was mixed with distilled water in a ratio of 1:4, and the mixture was left to stand at room temperature for approx. 30 min. Finally, the pH value was determined with a pH meter (SG2 Seven GoTM pH-Meter; Mettler-Toldo; Greifensee, Switzerland).

#### 2.3.6. *C. perfringens* in Sows’ Faeces Post Partum

The number of colony-forming units (CFU) of *C. perfringens* was determined in a rectal faecal sample taken from each sow immediately after birth (max. 10 h p.p.). For this purpose, a first dilution (1 g faeces in 9 mL sterile phosphate-buffered saline solution, PBS) was first made without prior enrichment and from this further decimal dilutions up to 10^−4^ were prepared. Exactly 0.1 mL was taken from each decimal dilution and added in duplicate to the surface of a selective culture medium for *C. perfringens* (so called NPC agar). The NPC agar was produced in a modified form at the Institute of Microbiology (University of Veterinary Medicine Hannover, Foundation) in accordance with Gad et al. [36]. The culture medium was based on a 5% sheep blood agar with the additives neomycin (200 μg/mL neomycin sulphate; research grade; Serva Electrophoresis GmbH; Heidelberg, Germany), polymyxin B (100 μg/mL Polymyxin B sulphate; BioChemica BC; AppliChem GmbH; Darmstadt, Germany) and cysteine (300 μg/mL LCysteine HCl-H2O; cryst. research grade; Serva Electrophoresis GmbH). The inoculated culture media were incubated at 37 °C for a total of 48 h in an anaerobic atmosphere (generated by AnaeroGen; Oxoid Deutschland GmbH; Wesel, Germany). The determination of the number of CFU of *C. perfringens* was performed analogously to the standard for *Campylobacter* spp. in a slightly modified form [37]. For this purpose, the number of colonies identified as *C. perfringens* was counted in both approaches and the mean value was calculated. However, only the plates on which 10–100 colonies had grown were considered. The classification of the *C. perfringens* cultures obtained with regard to the different toxin types was carried out randomly using a commercially available multiplex PCR kit (BACTOTYPE^®^ PCR Amplification Kit *C. perfringens*; Biotype Diagnostics GmbH; Dresden, Germany).

#### 2.3.7. Environmental Temperature

The experimental phase covered a period of seven months (June to December), which meant that the sows in the trial were exposed to different environmental temperatures.

Despite the alternating arrangement of the experimental runs, the FG-1 feeding runs were exposed to more heat than the FG-2 feeding animals. While the percentage of hot days (temperature in 24 h > 25 °C) in the peri partum period per sow in the FG-1 feeding runs was 23.6%, this was only 1.60% in the FG-2 feeding runs.

For this reason, the results of the studies are presented below separately for the two fibre groups with the respective control groups.

### 2.4. Statistical Analysis

Data were statistically analysed and recorded using SAS v. 7.1 (SAS Inst. Inc.; Cary, NC, USA) and Microsoft Excel 2016 (Microsoft Corp.; Redmond, WA, USA). Data were examined for their normal distribution using the Kolmogorov–Smirnov test and Shapiro–Wilk test. For group comparison (restrictively fed sows vs. ad libitum-fed sows; CG-1 vs. FG-1 and CG-2 vs. FG-2) for normally distributed data the t-test was applied and for non-normal data the unpaired two-samples Wilcoxon test. For the statistical evaluation of the *C. perfringens* bacterial counts, the values were previously logarithmized. The significance level was specified with 5%.

## 3. Results

### 3.1. Dry Matter Intake of the Different Feeding Groups

As described above, some sows could not be included in the statistics due to their function as foster sows. The daily amounts of feed intake in kg DM in relation to the day of birth ingested by sows in the control (CG-1 and CG-2) and fibre groups (FG-1 and FG-2) are shown in Table 5.

While the restrictively fed control animals ingested a relatively constant amount ~2.60 kg DM until birth, the feed intake of the animals in the experimental group supplemented with fibre-rich pellets ad libitum was at least 1.84 kg up to a maximum of 3.86 kg DM during these days and thus statistically significantly higher. A significant reduction in feed intake of the ad libitum-fed animals was only visible on the day of birth, but on this day the DM intake was approximately 1 kg higher than that of the restrictively fed control animals. Although the DM intake of fibre group 1 dropped below the level of the control group one day after birth—due to feed refusal of individual sows—and reached a low point of 2.96 ± 0.80 kg DM, the DM intake already increased on the second day post partum to the previously consumed amount ante partum. From the first day after birth onwards, the feed intake of the restrictively fed sows was gradually increased, thus gradually increasing the DM intake of the post-partum control group. All births proceeded without assistance and without irregularities. After birth until the second day post-partum, the DM intake did not differ statistically significantly between fibre group 1 and the control groups. Fibre group 2 consistently consumed significantly more feed than the control groups.

### 3.2. Daily Mean Feed Intake in kg DM during the Lactation Weeks Separated by Feeding Groups

**Figure 2:**
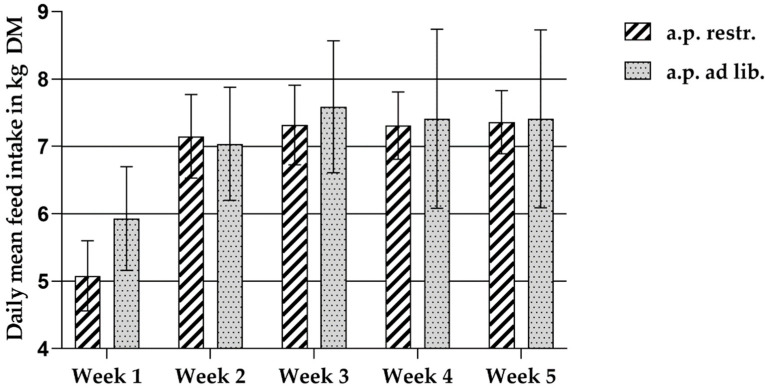
Mean values of the daily mean feed intake in kg DM during the lactation weeks separated by feeding groups (ante partum (a.p.) restrictively fed sows: n = 17; a.p. ad libitum-fed sows: n = 23 (FG-1, 2)).

The cross-fostering protocol (see Section 2.3.3) affected seven sows. In addition, one sow did not understand the functioning of the automatic feeder, so this sow was also excluded from the following evaluations (Figure 2). The fibre experimental groups (FG-1 and FG-2) were combined in the following evaluations. A potential effect of the respective fibre diets on feed intake during lactation was considered very unlikely. Thus, the evaluations of the examined parameters of ad libitum feeding in lactation were carried out on a total number of n = 40 sows (restrictively fed sows: n = 17, ad libitum fed sows: n = 23 (FG-1, 2)).

The feed intake on the days before birth has already been described in detail in Table 5, separated into fibre groups with the respective control groups. After birth, the animals of the ad libitum feeding group until the seventh day post partum showed a significantly higher feed intake compared to the restrictively fed control animals (Figure 2). From the second week of lactation onwards, the daily amount of ingested DM was almost identical in both groups. The significant increase in feed intake of the ad libitum-fed animals was clearly observed in the first days after birth when looking at the mean daily feed intake related to the lactation week. The mean DM intake of the ad libitum group was significantly higher in the first week of lactation and tended to be higher in the following weeks, except week 2, than in the control group. With the significantly higher feed intake after birth (Week 1) and the tendency of the ad libitum-fed group to have higher feed intake during each week of lactation, a higher mean DM intake of 7.02 ± 0.81 kg DM/day tended to be achieved by these animals throughout lactation than by the restrictively fed group (6.75 ± 0.44 kg DM/day) (Figure 2).

### 3.3. Mean Sows’ Body Weights (kg) in the Feeding Groups during Lactation

The sows of the ad libitum group tended to have higher weights at stabling compared to the restrictively fed control group. Until the second week, the sows of both feeding groups lost weight equally (Table 6).

Significantly lower body weights were observed in the restrictively fed sows at weaning after five weeks of lactation. The difference in mean body weight between the time of housing and weaning was also significantly higher in the control group compared to the ad libitum group. Body mass loss between 24 h after birth and stabling did not differ significantly between the two feeding groups.

### 3.4. Number of Piglets after Cross-Fostering Both Feeding Groups

Table 7 shows that there were almost no differences between the two feeding groups in terms of the number of piglets at the time of cross-fostering (c.f.) and weaning.

### 3.5. Fecal Analysis

At stabling and around the time of birth, the consistency of the fresh sow faeces was characterized in more detail using a scoring system (see Table 4).

All sows showed a similar faecal consistency when entering the farrowing pen (day −7). While the faeces of the sows in the a.p. restrictively fed sow group became increasingly harder towards the time of birth, a significantly smoother faecal consistency—with the exception of day 3 p.p.—was observed in the sows of the a.p. ad libitum-fed sow group until d5 after farrowing (Table 8)

### 3.6. Colony-Forming Units of C. perfringens in the Sows’ Faeces

The number of colony-forming units of *C. perfringens* in the faeces collected immediately post partum was statistically significantly lower in fibre group 1 (n = 8 sows) than in the faeces of control group 1 (n = 6 sows) (Table 9).

While an average of 3.02 ± 0.76 log10 CFU of *C. perfringens* could be detected in the faeces of the fibre-fed animals, this was about 10 times more in the faeces of the control animals with a number of 4.44 ± 0.99 log10 CFU. A total of 20 colonies typical for *C. perfringens*, which had been isolated from the faeces of 20 different sows each, were examined by PCR with regard to the toxin genes they contained. In all colonies examined, only the α-toxin-producing gene fragment cpa was detected. Thus, the colonies examined were bacteria of the *C. perfringens* type A species.

A statistically highly significant lower number of colony-forming units of *C. perfringens* could also be detected in the faeces of fibre group 2 (n = 12), which were collected immediately post partum, compared to the control group 2 (n = 13). Thus, the number of CFU in the faeces of the control animals at birth was approximately 100 times higher compared to the number of CFU in the faeces of the animals fed with fibre (4.84 ± 1.29 log10 CFU vs. 2.10 ± 1.50 log10 CFU). The mean number of CFUs of *C. perfringens* in faeces samples taken directly post partum did not differ significantly between the two groups receiving fibre (FG-1 and FG-2).

## 4. Discussion

### 4.1. Feed Intake in the Peripartal Period

In the days before birth, the average feed intake of the restrictively fed animals was about 2.6 kg DM. The additional daily voluntary intake amounts of the fibre/lactation diet mixture were 2.13 ± 1.15 kg DM (FG-1) and 3.14 ± 0.68 kg DM (FG-2). The sows in the ad libitum groups achieved high DM intake ante partum; the animals of FG-2 with 5.5–6.5 kg DM/d showed a 1 kg DM higher total feed intake compared to FG-1 in the first week of lactation. On the day of birth, both groups fed ad libitum showed a clear reduction in feed intake (consisting of a fibre/lactation diet mixture and the restrictively allocated lactation diet) to a quantity of approximately 3.7 kg DM, but on the second day post partum the previous ante partum feed intake was already reached. It was already observed in earlier studies that sows fed ad libitum already ante partum ingest high feed quantities, show a significantly reduced feed intake at birth and then return to the initial level within a short time [38,39]. While in a research study by Cools et al. [39] the ad libitum-fed group ante partum showed a similarly high feed intake as in later lactation (between 7–8 kg of lactation diet (891.9 g/kg DM)), the ad libitum-fed sows in this experiment reached the maximum feed intake only in lactation. However, it should be noted that in the study by Cools et al. [39], the sows received a commercial lactation diet (54.6 g CF/kg as-fed) from day 105 of gestation onward, whereas the sows in this trial were mainly fed fibre-rich pellets. Overall, the sows in the above-mentioned study by Cools et al. [39] ingested ante partum CF amounts between 383–437 g/d, based on dry matter; however, the sows in the present study showed an average of approximately 600 g CF/d, an approximately 33% higher CF intake. Such results indicate that sows, when given the opportunity, voluntarily consume large amounts of a supplemental feed rich in fibre without thereby changing the daily dietary energy supply. This may lead to a variety of beneficial effects. High amounts of fibre in the diet may have had an effect on the filling of the gastrointestinal tract and thus on the sows’ feelings of satiety. Similar results have been obtained in earlier studies. Kyriazakis and Emmans [40] found a linear decrease in feed intake when different fibre carriers such as wheat bran, citrus fibre or grass meal were fed to the animals, with an increasing water-binding capacity of the absorbed fibre and thus increasing gastrointestinal tract filling. De Leeuw et al. [41] also reported a saturation in sows induced by fibre intake. Bergeron et al. [14] observed that sows fed a very high fibre diet (23% CF as-fed) during gestation spent significantly more time eating than animals in the control group. Therefore, giving access to additional high fibre sources during gestation is a simple method to promote satiety and reduce stereotypical behaviour by increasing the time spent on feeding-related behaviours [14,40].

In our study, the sows of the two fibre groups showed no evidence of constipation and associated birth disorders such as an increased farrowing duration or an increased need for farrowing assistance compared to the controls. This result is in line with the findings of Marti et al. [42], who also evaluated the effects of ad libitum feeding on the performance of sows during farrowing. Although the authors did not feed the sows a high-fibre diet but a conventional gestation lactation diet (5% CF as-fed (overall)) ad libitum, they did not observe any negative influences of this feeding strategy on the farrowing process. Oliviero et al. [17] demonstrated that doubling the fibre content of the gestation diet (up to 7% CF as-fed) helped the intestine by reducing the occurrence of prolonged constipation during the delicate phase around farrowing and early lactation. They mentioned that high amounts of fibre in the diet positively affected the intestinal activity of sows, characterized by significantly higher faecal score values compared to the controls, that consumed a 3.8% fibre diet.

The overall significantly lower feed intake of the animals in fibre group 1 in the peripartal period that were exposed to more heat days (23.6%) compared to the animals in the fibre group 2 (1.60%) confirms the results of many other studies [43,44,45,46,47,48]. In these studies, too, lower feed intake was observed with rising temperatures in the farrowing pen. While the feed intake of fibre group 2 increased rapidly after birth, it decreased even more in fibre group 1 on the first day after birth. It can therefore be assumed that high ambient temperatures, especially around the time of birth, have a particularly negative effect on the feed intake of the affected sows.

### 4.2. Feed Intake during Lactation

In the ad libitum-fed sow group, a significantly higher feed intake was observed in the first week of lactation and in the following weeks, except for week 2, with a tendency towards higher feed intake compared to the restrictively fed control group. Accordingly, the feed intake during lactation was slightly higher in the ad libitum group (6.75 ± 0.44 kg DM vs. 7.02 ± 0.81 kg DM). This supports the observations of other studies which report higher feed intake of ad libitum-fed sows during lactation [38,39,49,50]. The overall lower feed intake of the restrictively fed sows that were fed almost ad libitum from day 10 p.p. onwards shows that these sows do not seem to be able to make up for the missing feed quantities of the first lactation week by compensatory higher feed intake in the following lactation weeks. This finding was also made by other authors [4,42,51] and Neil [38], who showed in his study that the earlier the sows were given ad libitum feed, the higher their feed intake was during lactation. However, in addition to the ad libitum supply of the lactation diet, the higher feed intake of the ad libitum feeding group could also be due to the fact that these animals had an additional ration with high fibre content available to them in the peripartal period. A study by Quesnel et al. [19] showed a 0.94 kg daily higher lactation diet intake in sows fed a mixture of sunflower meal, sugar beet pulp, wheat bran and soybean hulls during pregnancy. Other authors also observed higher feed intake during lactation if high fibre rations were previously given [20,52,53,54,55]. Farmer et al. [56] see the reason for this phenomenon in the fact that the gastrointestinal tract of these animals had already become accustomed to large amounts of feed due to the voluminous raw fibre masses, whereas in animals without added fibre they must first adapt to such amounts of feed.

### 4.3. Body Mass Development of the Sows

In all sows, body mass melted during lactation. Thus, despite the high feed intake and the fact that during lactation the daily energy intake of lactating sows as specified by Kamphues et al. [2] with a litter gain of 2–3 kg per day of 90–98 MJ ME was achieved with an average of 99.2 MJ ME in the control group and 103 MJ ME in the ad libitum group, an energy deficit must have occurred. However, the sows in the ad libitum group entered the farrowing pen with the highest body weight and greatest backfat thickness and lost proportionally less body mass over the entire lactation, so that they had a significantly higher body weight and body condition score at weaning. This contradicts the findings of many other studies reporting elevated mobilization of back fat throughout lactation when sows had greater fat reserves at farrowing [4,39,57,58]. However, the thickness of the fat layer determined at the time of birth seems to be decisive for the further course of lactation. Against this background that negative effects in lactation are only reported for a backfat thickness of 22–25 mm [39,59], even the mean backfat thickness of the ad libitum-fed sows of only approximately 17 mm might not have been sufficient to exert negative influences on feed intake and consequently the loss of backfat. While the sows of the ad libitum-fed group gained about 0.5 mm backfat thickness in the period between housing and the first day p.p., this amount of backfat was already melted down by the restrictively fed sows in this period, so that the backfat thickness at the beginning of lactation differed between the two feeding groups by a total of about 1.2 mm. Similar observations were made by Cools et al. [39]. With ad libitum feeding of a commercial lactation diet from 7 days before birth, these sows also had an increase of on average 0.6 ± 2.1 mm of backfat. Simultaneously, this study also found a loss of 0.4 ± 1.9 mm in the restrictively fed sows. Against this background, it appears that the energy and nutrient supply in the case of the restrictive feeding of the lactation diet seems insufficient to meet the sows’ requirements even before birth. As a result, these sows suffer from a loss of body substance already at the beginning of lactation, which reduces the energy available to the sows for lactation. With an overall backfat loss of 3.59 mm, the sows in the restrictively fed group lost about 0.5 mm more back fat compared to the ad libitum group. Nevertheless it should be noted that a backfat loss of about 3 mm is observed in most studies already at a suckling period of only 3–4 weeks [60].

As the mean feed intake was almost identical in both groups from the second week of lactation onwards, the significantly different energy and nutrient intake in the first week of lactation must not only have led to an increase in backfat thickness but also seemed to have had a longer-lasting modulating effect on the metabolism of these sows. Other authors have also found a reduction in the melting of body substance with increasing feed intake during lactation [39,60,61]. Neil [38] also observed that the faster sows were fed ad libitum after birth, the lower the backfat losses were during lactation. Thus, an ad libitum feeding regime based on high amounts of fibre seems to relieve sows in lactation despite high milk yield.

### 4.4. Content of C. perfringens ssp. in Feces Post Partum

While the number of CFUs of *C. perfringens* in the sows’ faeces taken immediately after giving birth in both control groups was approximately 4.5 log_10_ CFU/g faeces, the number of CFU in the faeces of the two fibre groups was reduced by a factor of 10 and 100 (FG-1: 3.02 ± 0.76 log_10_ CFU/g faeces; FG-2: 2.10 ± 1.50 log_10_ CFU/g faeces), respectively. In other studies a reduction in the number of *Clostridia* in pigs’ faeces was also achieved by feeding them a diet rich in fibre [62,63,64]. Feeding, for example, Jerusalem artichoke or clover grass silage to pregnant sows led to a tenfold reduction in the number of *Clostridia* (from initial values of 4 to 5 log_10_ CFU/g faeces to values of 3 to 3.5 log_10_ CFU/g faeces) [63]. Here, the content of fermentable fibre seemed to be important. These effects were observed in particular when easily fermentable polysaccharides such as the prebiotic inulin or konjac flour—the ground rootstock of the devil’s tongue, which consists mainly of glucomannans—were used [30,62,64]. If, on the other hand, straw was fed, no positive effect on the number of *Clostridia* in faeces was observed. In vitro studies by May et al. [65] also came to similar results. The authors attributed the reduction in the number of *C. perfringens* in sows’ faeces with the intake of fermentable fibre to the increased formation of short-chain fatty acids and the resulting pH reduction (depending on the fibre source, up to pH 4.17 (in vitro)) [65]. Because the present study also found a significantly lower pH value in the faeces of the animals fed a diet rich in fibre, and because other studies also point to a changed microbiological composition of the intestinal flora at low pH values [66,67], the changed intestinal environment caused by the fibre-rich diet may have minimised the proliferation of *C. perfringens.* This would also explain the significantly lower *C. perfringens* count in the faeces of FG-2. Due to the higher uptake of fibre pellet 2 and consequently of bacterially fermentable substance, the pH in the faeces of these animals was lowered more strongly (6.42 ± 0.39 vs. 6.35 ± 0.53 24 h p.p.). Within the framework of the randomly conducted molecular biological examination of the bacterial cultures isolated from the faeces, the α-toxin could be detected as the only major toxin. Thus, the cultures analysed were bacteria of the species *C. perfringens* type A. Although this bacterial species also occurs regularly in healthy animals [26], *C. perfringens* type A is nevertheless quite capable of causing diarrhoea, especially in suckling pigs [24,25]. Despite the significantly higher concentrations of *C. perfringens* in the faeces of the restrictively fed control animals, no adverse health effects were found in the sows nor the piglets. When comparing our findings with earlier studies [26,63,68], it is striking that the amount of *C. perfringens* of 10^4^/g faeces of the restrictively fed sows is also found in healthy fattening pigs or pregnant and lactating sows. Therefore, the absence of clinical symptoms in this study does not seem surprising. Nevertheless, a significant reduction in the germ count by supplementing a feed rich in fibre deserves special attention, especially since the newborn piglets come into direct contact with the sow’s faeces immediately after birth. Sows’ faeces are an important source of infection for diarrhoea caused by *C. perfringens* type A in suckling pigs [24,69]. Especially for farms with *C. perfringens* diseases, this observation could be useful as an aid in preventing further cases of disease.

## 5. Conclusions

In conclusion, increased feed intake of ad libitum diets rich in fibre ante partum did not interfere with the farrowing process, nor did it increase the incidence of any associated disorders such as post partum dysgalactia syndrome. Furthermore, peripartal ad libitum-fed sows showed higher DM intakes throughout lactation, which had beneficial effects on the performance of the sows. Due to the high fibre intake, the excretion of *C. perfringens* via sows’ faeces at parturition could be significantly reduced, which could be a helpful tool in reducing the risk of neonatal diarrhoea caused by *C. perfringens*. Overall, it can be concluded that the additional high-fibre feeding has positive effects on the health and performance of sows.

## Figures and Tables

**Table 1:** Composition of the commercial lactation diet according to the declaration.

Components	%
wheat	39.00
barley	20.00
soy extraction meal	10.00
wheat flour	8.00
wheat bran	7.50
wheat semolina bran	4.00
whole soybean	3.20
rapeseed cake	3.00
oat husk bran	3.00
calcium carbonate	1.75
sodium chloride	0.40
monocalcium phosphate	0.15

**Table 2:** Composition of fibre pellet 1 and fibre pellet 2 according to the declaration.

Components	Fibre Pellet 1 (%)	Fibre Pellet 2 (%)
barley meal	49.00	49.00
soybean hulls (pressure-hydrothermally treated)	49.00	24.75
oat hulls		20.00
soybean meal, extr. (44% CP)		4.00
molasses	1.50	1.50
sodium chloride	0.50	0.50
calcium carbonate		0.25

**Table 3:** Chemical composition of the lactation diet fibre pellet 1 and fibre pellet 2.

Ingredients		Lactation Diet	Fibre Pellet 1	Fibre Pellet 2
crude protein	g/kg DM	190	119	125
crude fat		40.8	29.5	24.5
crude fibre		48.3	197	179
lysine		11.3	5.97	6.15
methionine		2.86	2.18	1.78
calcium		11.0	4.07	5.18
phosphorus		6.62	2.29	2.96
neutral detergent fibre		186	426	431
acid detergent fibre		56.8	253	230
Energy *	MJ ME/kg DM	14.7	10.1	10.0

* Calculated in accordance with GFE [33] for lactation diets and in accordance with Kirchgessner and Roth [34] for fibre pellets.

**Table 4:** Scoring system for sows’ faeces according to Oliviero et al. [17].

Symptoms	Score
absence of faeces	0
dry and pellet-shaped	1
between dry and normal	2
normal and soft, but firm and well formed	3
between normal and wet, still formed but not firm	4
wet, unformed and liquid	5

**Table 5:** Dry matter intake in kg/d and sow group in the peripartal period (d0 = parturition) in the four feeding groups.

Day	CG-1		FG-1		CG-2		FG-2	
	Restrictively Fed Sows		Ad Libitum- Fed Sows		Restrictively Fed Sows		Ad Libitum- Fed Sows	
	n *	x	n *	x	n *	x	n *	x
−6	6	2.60 ^a^ ± 0.32	8	5.58 ^b^ ± 1.68	5	2.57 ^a^ ± 0.05	5	5.65 ^b^ ± 1.40
−5	8	2.58 ^a^ ± 0.33	11	4.59 ^b^ ± 1.35	11	2.63 ^a^ ± 0.24	7	5.71 ^c^ ± 0.90
−4	9	2.55 ^a^ ± 0.31	12	4.80 ^b^ ±1.00	13	2.63 ^a^ ± 0.23	11	6.49 ^c^ ± 1.76
−3	9	2.55 ^a^ ± 0.31	12	4.83 ^b^ ± 1.48	13	2.63 ^a^ ± 0.23	12	6.16 ^c^ ± 1.43
−2	10	2.58 ^a^ ± 0.31	12	4.56 ^b^ ± 1.41	13	2.63 ^a^ ± 0.23	12	5.94 ^c^ ± 1.22
−1	10	2.58 ^a^ ± 0.31	12	4.42 ^b^ ± 0.87	13	2.63 ^a^ ± 0.23	12	5.29 ^c^ ±1.02
0	10	2.58 ^a^ ± 0.31	12	3.67 ^b^ ± 1.50	13	2.63 ^a^ ± 0.23	12	3.73 ^b^ ± 1.42
1	10	3.09 ^a^ ± 0.51	12	2.96 ^a^ ± 0.80	13	2.90 ^a^ ± 0.39	12	3.99 ^c^ ±0.96
2	10	3.84 ^a^ ± 1.11	12	4.54 ^a^ ± 1.35	13	3.77 ^a^ ± 0.93	12	5.80 ^c^ ± 1.22

* Not all sows farrowed on the same day, so a different number of sows were included in the statistical evaluation in days −6 to −3 depending on the farrowing day of each sow (see Section 2.3.1). ^a,b,c^ averages differ significantly within a line (*p* < 0.05).

**Table 6:** Mean values of the sows’ body masses (kg) and backfat thicknesses (mm) in the feeding groups during lactation.

Day	a.p.* Restrictively Fed Sows			a.p.* Ad Libitum-Fed Sows		
	n	Body Mass (kg)	Backfat (mm)	n	Body Mass (kg)	Backfat (mm)
Stabling	17	279 ^a^ ± 36.3	16.9 ± 3.24	23	288 ^a^ ± 27.8	17.1 ± 4.21
Day 1 p.p.*	17	263 ^a^ ± 32.9	16.4 ± 3.36	23	281 ^a^ ± 33.4	17.6 ± 4.38
Day 14 p.p.*	10	261 ^a^ ± 31.8	15.4 ± 3.12	14	281 ^a^ ± 32.5	16.8 ± 3.85
Day of removal	17	240 ^a^ ± 31.9	12.9 ± 3.29	23	261 ^b^ ± 35.0	14.5 ± 3.33
Diff_Lac	17	−23.4 ^a^ ± 12.5	−3.59 ± 1.84	23	−19.7 ^a^ ± 11.9	−3.09 ± 1.98
Diff_total	17	−39.1 ^a^ ± 15.5	−4.04 ^A^ ± 1.74	23	−26.8 ^b^ ± 13.2	−2.52 ^B^ ± 2.04

* a.p. = ante partum, p.p. = post partum; ^a,b^ averages differ significantly within a line, body mass (*p* < 0.05); ^A,B^ averages differ significantly within a line, backfat (*p* < 0.05).

**Table 7:** Mean values of the number of piglets after cross-fostering (c.f.) for weaning and losses after c.f. of both feeding groups.

	a.p.* Restrictively Feed Sows (n = 17)	a.p.* Ad Libitum-Fed Sows (n = 23)	
			*p*-Value
piglets after c.f.	13.4 ± 1.11	13.0 ± 1.60	0.57
weaned piglets	12.2 ± 2.53	11.9 ±1.90	0.42
Losses after c.f.	−1.18 ± 1.78	−1.13 ± 1.10	0.53

* a.p. = ante partum.

**Table 8:** Mean values of the faecal score of the ante partum (a.p.) restrictively fed sows and the a.p. ad libitum-fed sows.

Day	a.p.*^1^ Restrictively Fed Sows (n = 23)	a.p.*^1^ Ad Libitum-Fed Sows (n = 25)
−7	2.95 ^a^ ± 0.50	2.94 ^a^ ± 0.11
−4	2.15 ^b^ ± 0.63	3.35 ^a^ ± 0.63
−2	1.55 ^b^ ± 0.64	3.17 ^a^ ± 0.36
−1	1.45 ^b^ ± 0.72	3.48 ^a^ ± 0.68
0 *^2^	1.05 ^b^ ± 0.72	2.50 ^a^ ± 0.90
1	1.50 ^b^ ± 0.53	2.59 ^a^ ± 1.07
2	1.85 ^b^ ± 0.47	2.63 ^a^ ± 0.35
3	2.58 ^a^ ± 0.93	2.84 ^a^ ± 0.50
5	2.33 ^b^ ± 0.99	2.94 ^a^ ± 0.45

^a,b^ averages differ significantly within a line body mass (*p* < 0.05); *^1^ a.p. = ante partum; *^2^ 0 = day of birth.

**Table 9:** Average number of colony-forming units of *C. perfringens* (log_10_ CFU/g) in the faeces of the sows in the control and fibre groups taken directly post partum.

CG-1 (n = 6)		FG-1 (n = 8)		CG-2 (n = 13)		FG-2 (n = 12)	
pH	log10 CFU	pH	log10 CFU	pH	log10 CFU	pH	log10 CFU
7.20 ^a^ ± 0.38	4.44 ^A^ ± 0.99	6.42 ^b^ ± 0.39	3.02 ^B^ ± 0.76	7.16 ^a^ ± 0.37	4.8 ^A^ ± 1.29	6.35 ^b^ ± 0.53	2.10 ^B^ ± 1.50

^a,b,A,B^ averages differ significantly within a line (*p* < 0.05).

## Data Availability

The data presented in this study are available in this manuscript.

## References

[1] Strathe A.V., Bruun T.S., Hansen C.F. (2017). Sows with high milk production had both a high feed intake and high body mobilization. Animals.

[2] Kamphues J., Wolf P., Coenen M., Edder K., Iben C., Kienzle E., Liesegang A., Männer K., Zebeli Q., Zentek J. (2014). Supplemente zur Tierernährung: Für Studium und Praxis.

[3] Eissen J.J., Kanis E., Kemp B. (2000). Sow factors affecting voluntary feed intake during lactation. Livest. Prod. Sci..

[4] Vignola M. Sow feeding management during Lactation. Proceedings of the London Swine Conference-Tools of Trade.

[5] King R., Dunkin A. (1986). The effect of nutrition on the reproductive performance of first-litter sows 3. The response to graded increases in food intake during lactation. Anim. Sci..

[6] Theil P.K., Farmer C. (2015). Transition feeding of sows. The Gestating and Lactating Sow.

[7] Hansen A.V., Lauridsen C., Sørensen M.T., Knudsen K.E.B., Theil P.K. (2012). Effects of nutrient supply, plasma metabolites, and nutritional status of sows during transition on performance in the next lactation1. J. Anim. Sci..

[8] Feyera T., Theil P.K. (2017). Energy and lysine requirements and balances of sows during transition and lactation: A factorial approach. Livest. Sci..

[9] Schneider H. (1991). Placental transport function. Reprod. Fertil. Dev..

[10] Theil P.K., Sejrsen K., Hurley W.L., Labouriau R., Thomsen B., Sørensen M.T. (2006). Role of suckling in regulating cell turnover and onset and maintenance of lactation in individual mammary glands of sows. J. Anim. Sci..

[11] Feyera T., Pedersen T.F., Krogh U., Foldager L., Theil P.K. (2018). Impact of sow energy status during farrowing on farrowing kinetics, frequency of stillborn piglets, and farrowing assistance. J. Anim. Sci..

[12] Nielsen S.E., Feyera T., Skovmose S.J.W., Krogh U., Eskildsen M., Theil P.K. (2021). Intravenous infusion of glucose improved farrowing performance of hyperprolific crossbred sows. J. Anim. Sci..

[13] Gourley K.M., Swanson A.J., DeRouchey J.M., Tokach M.D., Dritz S.S., Goodband R.D., Woodworth J.C. (2020). Effects of increased lysine and energy feeding duration prior to parturition on sow and litter performance, piglet survival, and colostrum quality. J. Anim. Sci..

[14] Bergeron R., Bolduc J., Ramonet Y., Meunier-Salaün M.C., Robert S. (2000). Feeding motivation and stereotypies in pregnant sows fed increasing levels of fibre and/or food. Appl. Anim. Behav. Sci..

[15] Meunier-Salaün M.C., Edwards S.A., Robert S. (2001). Effect of dietary fibre on the behaviour and health of the restricted fed sow. Anim. Feed Sci. Technol..

[16] Lindberg J.E. (2014). Fiber effects in nutrition and gut health in pigs. J. Anim. Sci. Biotechnol..

[17] Oliviero C., Kokkonen T., Heinonen M., Sankari S., Peltoniemi O. (2009). Feeding sows with high fibre diet around farrowing and early lactation: Impact on intestinal activity, energy balance related parameters and litter performance. Res. Vet. Sci..

[18] Feyera T., Højgaard C.K., Vinther J., Bruun T.S., Theil P.K. (2017). Dietary supplement rich in fiber fed to late gestating sows during transition reduces rate of stillborn piglets. J. Anim. Sci..

[19] Quesnel H., Meunier-Salaün M.C., Hamard A., Guillemet R., Etienne M., Farmer C., Dourmad J.Y., Père M.C. (2009). Dietary fiber for pregnant sows: Influence on sow physiology and performance during lactation. J. Anim. Sci..

[20] Agyekum A.K., Columbus D.A., Farmer C., Beaulieu A.D. (2019). Effects of supplementing processed straw during late gestation on sow physiology, lactation feed intake, and offspring body weight and carcass quality1. J. Anim. Sci..

[21] Cummings D.E., Overduin J. (2007). Gastrointestinal regulation of food intake. J. Clin. Investig..

[22] Macpherson A.J., de Agüero M.G., Ganal-Vonarburg S.C. (2017). How nutrition and the maternal microbiota shape the neonatal immune system. Nat. Rev. Immunol.

[23] Richards J.D., Gong J., Lange C.F.M.d. (2005). The gastrointestinal microbiota and its role in monogastric nutrition and health with an emphasis on pigs: Current understanding, possible modulations, and new technologies for ecological studies. Can. J. Anim. Sci..

[24] Songer J.G., Uzal F.A. (2005). Clostridial enteric infections in pigs. J. Vet. Diagn. Investig..

[25] Uzal F.A., Songer J.G., Zimmerman J.J., Karriker L.A., Ramirez A., Schwartz K.J., Stevenson G.W., Zhang J. (2019). Clostridial Diseases. Diseases of Swine.

[26] Chan G., Farzan A., Soltes G., Nicholson V.M., Pei Y., Friendship R., Prescott J.F. (2012). The epidemiology of Clostridium perfringens type A on Ontario swine farms, with special reference to cpb2-positive isolates. BMC Vet. Res..

[27] Le Bourgot C., Ferret-Bernard S., Le Normand L., Savary G., Menendez-Aparicio E., Blat S., Appert-Bossard E., Respondek F., Le Huërou-Luron I. (2014). Maternal short-chain fructooligosaccharide supplementation influences intestinal immune system maturation in piglets. PLoS ONE.

[28] Laskowska E., Jarosz Ł., Grądzki Z. (2019). Effect of multi-microbial probiotic formulation bokashi on pro- and anti-inflammatory cytokines profile in the serum, colostrum and milk of sows, and in a culture of polymorphonuclear cells isolated from colostrum. Probiotics Antimicrob. Proteins.

[29] Bernardino T., Tatemoto P., Morrone B., Rodrigues P.H.M., Zanella A.J. (2016). Piglets Born from Sows Fed High Fibre Diets during Pregnancy Are Less Aggressive Prior to Weaning. PLoS ONE.

[30] Li H., Ma L., Zhang L., Liu N., Li Z., Zhang F., Liu X., Ma X. (2021). Dietary inulin regulated gut microbiota and improved neonatal health in a pregnant sow model. Front. Nutr..

[31] Naumann C., Bassler R. (1976). Methoden der landwirtschaftlichen Forschungs-und Untersuchungsanstalt, Biochemische. Untersuchung von Futtermitteln. Methodenbuch III (einschließlich der achten Ergänzungen. bis 2012).

[32] Visscher C., Mischok J., Sander S., Schmicke M., Peitzmeier E.-U., von dem Busche I., Rohn K., Kamphues J. (2018). Nutrient digestibility, organ morphometry and performance in vaccinated or non-vaccinated Lawsonia intracellularis infected piglets. BMC Vet. Res..

[33] GfE (2008). Prediction of metabolisable energy of compound feeds for pigs. Proc. Soc. Nutr. Physiol..

[34] Kirchgessner M., Roth F. (1983). Schätzgleichungen zur Ermittlung des energetischen Futterwertes von Mischfuttermitteln für Schweine. Z. Tierphysiol. Tierernährung Futterm..

[35] Müller S., Polten S. (2004). Vergleichsuntersuchungen zur Ultraschall-Speckdickenmessung beim Schwein im Rahmen der Eigenleistungsprüfung. Arch. Anim. Breed..

[36] Gad W., Hauck R., Krueger M., Hafez H. (2011). Prevalence of Clostridium perfringens in commercial turkey and layer flocks. Arch. Geflugelkd..

[37] (2006). Microbiology of Food and Animal Feeding Stuffs-Horizontal Method for Detection and Enumeration of Campylobacter spp.: Part 2: Colony-Count Technique.

[38] Neil M. (1996). Ad libitum lactation feeding of sows introduced immediately before, at, or after farrowing. Anim. Sci..

[39] Cools A., Maes D., Decaluwé R., Buyse J., van Kempen T.A.T.G., Liesegang A., Janssens G.P.J. (2014). Ad libitum feeding during the peripartal period affects body condition, reproduction results and metabolism of sows. Anim. Reprod. Sci..

[40] Kyriazakis I., Emmans G.C. (1995). The voluntary feed intake of pigs given feeds based on wheat bran, dried citrus pulp and grass meal, in relation to measurements of feed bulk. Br. J. Nutr..

[41] de Leeuw J.A., Bolhuis J.E., Bosch G., Gerrits W.J. (2008). Effects of dietary fibre on behaviour and satiety in pigs. Proc. Nutr. Soc..

[42] Martí L., Latorre M., Álvarez-Rodríguez J. (2019). Does ad libitum feeding during the peri-partum improve the sow feed intake and performances?. Animals.

[43] Koketsu Y., Dial G.D., Pettigrew J.E., Marsh W.E., King V.L. (1996). Characterization of feed intake patterns during lactation in commercial swine herds. J. Anim. Sci..

[44] Prunier A., De Braganca M.M., Le Dividich J. (1997). Infl.luence of high ambient temperature on performance of reproductive sows. Livest. Prod. Sci..

[45] Quiniou N., Noblet J. (1999). Influence of high ambient temperatures on performance of multiparous lactating sows. J. Anim. Sci..

[46] Bergsma R., Hermesch S. (2012). Exploring breeding opportunities for reduced thermal sensitivity of feed intake in the lactating sow. J. Anim. Sci..

[47] Williams A.M., Safranski T.J., Spiers D.E., Eichen P.A., Coate E.A., Lucy M.C. (2013). Effects of a controlled heat stress during late gestation, lactation, and after weaning on thermoregulation, metabolism, and reproduction of primiparous sows. J. Anim. Sci..

[48] Bjerg B., Brandt P., Pedersen P., Zhang G. (2020). Sows’ responses to increased heat load-A review. J. Therm. Biol..

[49] Stahly T., Cromwell G., Simpson W. (1979). Effects of full vs. restricted feeding of the sow immediately postpartum on lactation performance. J. Anim. Sci..

[50] Moser R., Cornelius S., Pettigrew J., Hanke H., Heeg T., Miller K. (1987). Influence of postpartum feeding method on performance of the lactating sow. Livest. Prod. Sci..

[51] Aherne F.X., Williams I.H. (1992). Nutrition for optimizing breeding herd performance. Vet. Clin. N. Am. Food Anim. Pract..

[52] Danielsen V., Vestergaard E.M. (2001). Dietary fibre for pregnant sows: Effect on performance and behaviour. Anim. Feed Sci. Technol..

[53] Veum T.L., Crenshaw J.D., Crenshaw T.D., Cromwell G.L., Easter R.A., Ewan R.C., Nelssen J.L., Miller E.R., Pettigrew J.E., Ellersieck M.R. (2009). The addition of ground wheat straw as a fiber source in the gestation diet of sows and the effect on sow and litter performance for three successive parities. J. Anim. Sci..

[54] Guillemet R., Dourmad J.Y., Meunier-Salaün M.C. (2006). Feeding behavior in primiparous lactating sows: Impact of a high-fiber diet during pregnancy. J. Anim. Sci..

[55] Shang Q., Liu H., Liu S., He T., Piao X. (2019). Effects of dietary fiber sources during late gestation and lactation on sow performance, milk quality, and intestinal health in piglets1. J. Anim. Sci..

[56] Farmer C., Robert S., Matte J.J. (1996). Lactation performance of sows fed a bulky diet during gestation and receiving growth hormone-releasing factor during lactation. J. Anim. Sci..

[57] Dourmad J.-Y. (1991). Effect of feeding level in the gilt during pregnancy on voluntary feed intake during lactation and changes in body composition during gestation and lactation. Livest. Prod. Sci..

[58] De Rensis F., Gherpelli M., Superchi P., Kirkwood R. (2005). Relationships between backfat depth and plasma leptin during lactation and sow reproductive performance after weaning. Anim. Reprod. Sci..

[59] Whittemore C. (1996). Nutrition reproduction interactions in primiparous sows. Livest. Prod. Sci..

[60] Koketsu Y., Dial G.D., Pettigrew J.E., Xue J., Yang H., Lucia T. (1998). Influence of lactation length and feed intake on reproductive performance and blood concentrations of glucose, insulin and luteinizing hormone in primiparous sows. Anim. Reprod. Sci..

[61] Maes D., Janssens G., Delputte P., Lammertyn A., de Kruif A. (2004). Back fat measurements in sows from three commercial pig herds: Relationship with reproductive efficiency and correlation with visual body condition scores. Livest. Prod. Sci..

[62] Patterson J.K., Yasuda K., Welch R.M., Miller D.D., Lei X.G. (2010). Supplemental dietary inulin of variable chain lengths alters intestinal bacterial populations in young pigs. J. Nutr..

[63] Schubbert A., Werner C., Sundrum A. (2010). Raufuttergabe an Sauen als Präventivmaßnahme Gegen Sauen-und Ferkelerkrankungen.

[64] Tan C., Wei H., Sun H., Long G., Ao J., Jiang S., Peng J. (2015). Effects of supplementing sow diets during two gestations with konjac flour and Saccharomyces boulardii on constipation in peripartal period, lactation feed intake and piglet performance. Anim. Feed Sci. Technol..

[65] May T., Mackie R., Fahey G., Cremin J., Garleb K. (1994). Effect of fiber source on short-chain fatty acid production and on the growth and toxin production by Clostridium difficile. Scand J. Gastroenterol..

[66] Wenk C. (2001). The role of dietary fibre in the digestive physiology of the pig. Anim. Feed Sci. Technol..

[67] Louis P., Scott K.P., Duncan S.H., Flint H.J. (2007). Understanding the effects of diet on bacterial metabolism in the large intestine. J. Appl. Microbiol..

[68] Tschirdewahn B., Notermans S., Wernars K., Untermann F. (1991). The presence of enterotoxigenic Clostridium perfringens strains in faeces of various animals. Int. J. Food Microbiol..

[69] Allaart J.G., van Asten A.J., Gröne A. (2013). Predisposing factors and prevention of Clostridium perfringens-associated enteritis. Comp. Immunol. Microbiol. Infect. Dis..

